# Bioavailability of an R-α-Lipoic Acid/γ-Cyclodextrin Complex in Healthy Volunteers

**DOI:** 10.3390/ijms17060949

**Published:** 2016-06-15

**Authors:** Naoko Ikuta, Hinako Okamoto, Takahiro Furune, Yukiko Uekaji, Keiji Terao, Ryota Uchida, Kosuke Iwamoto, Atsushi Miyajima, Takashi Hirota, Norihiro Sakamoto

**Affiliations:** 1 Graduate School of Medicine, Kobe University, Kobe 650-0017, Japan; naoko.ikuta@people.kobe-u.ac.jp (N.I.); hinako.okamoto@cyclochem.com (H.O.); keiji.terao@cyclochem.com (K.T.); 2 CycloChem Bio Co., Ltd., Kobe 650-0047, Japan; takahiro.furune@cyclochem.com (T.F.); yukiko.uekaji@cyclochem.com (Y.U.); 3 Department of Biopharmaceutics, Faculty of Pharmaceutical Science, Tokyo University of Science, Chiba 278-8510, Japan; j3b13702@ed.tus.ac.jp (R.U.); gan.ln284@gmail.com (K.I.); miyajima@my-pharm.ac.jp (A.M.); hirotas5@rs.noda.tus.ac.jp (T.H.)

**Keywords:** R-α-lipoic acid, cyclodextrin, bioavailability, pharmacokinetics, healthy volunteers, clinical study, oral administration

## Abstract

R-α-lipoic acid (R-LA) is a cofactor of mitochondrial enzymes and a very strong antioxidant. R-LA is available as a functional food ingredient but is unstable against heat or acid. Stabilized R-LA was prepared through complexation with γ-cyclodextrin (CD), yielding R-LA/CD. R-LA/CD was orally administered to six healthy volunteers and showed higher plasma levels with an area under the plasma concentration-time curve that was 2.5 times higher than that after oral administration of non-complexed R-LA, although the time to reach the maximum plasma concentration and half-life did not differ. Furthermore, the plasma glucose level after a single oral administration of R-LA/CD or R-LA was not affected and no side effects were observed. These results indicate that R-LA/CD could be easily absorbed in the intestine. In conclusion, γ-CD complexation is a promising technology for delivering functional but unstable ingredients like R-LA.

## 1. Introduction

**Figure 1:**
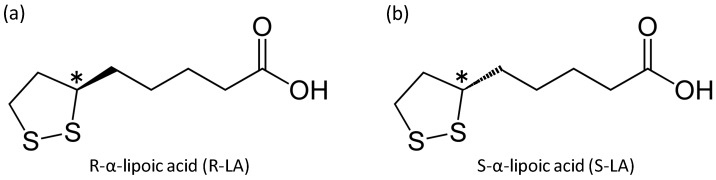
Chemical structure of R-α-lipoic acid (**a**) and S-α-lipoic acid (**b**). The chiral center is marked with an asterisk (*).

α-Lipoic acid (LA) has both a hydrophobic and hydrophilic part, and a chiral center at the C6 carbon in its 1,2-dithiolane ring; therefore, there are two enantiomers, R-α-lipoic acid (R-LA) and S-LA, of which R-LA is the naturally occurring compound (Figure 1). R-LA exists in the mitochondria and functions as a cofactor of mitochondrial enzymes. R-LA plays an important role in energy expenditure and also as a very powerful antioxidant. R-LA can restore other antioxidants such as ubisemiquinone or glutathione in the body [1]. It seems that both R-LA and S-LA have different potencies. R-LA is more potent than the S-LA in its efficacy to stimulate glucose uptake in L6 myotubes, and also to increase insulin-stimulated glucose uptake in obese Zucker rats [2]. R-LA is bound to proteins as a cofactor of such as pyruvate dehydrogenase and α-ketoglutaric dehydrogenase and α-ketoacid dehydrogenases in mitochondria. [3]. As well as protein-bound LA, free LA seems to be a promising ingredient for nutraceuticals and pharmaceuticals because of its fundamental role in metabolism and antioxidant properties [4]. Antioxidant properties of LA consist of the following: (1) its ability to scavenge reactive oxygen species (ROS) directly; (2) its strong redox potential to regenerate other endogenous antioxidants, such as glutathione and vitamins E and C; and (3) its metal-chelating activity, thus lowering ROS production. [5].

Clinical trials have shown that LA is effective for the treatment of diabetes or diabetic complications (such as diabetic neuropathy) [6,7,8,9,10,11,12,13,14], Alzheimer’s disease [15,16,17], liver diseases [18], and peripheral artery disease [19]. Furthermore, LA had effects on body weight in obese subjects [20,21,22]. Racemic LA, a mixture of the same amount of R-LA and S-LA, is widely available as a functional food or medical supplement for anti-aging, anti-diabetic, or anti-obesity purposes. R-LA is the bioactive enantiomer and is biosynthesized, but S-LA is not. R-LA is unstable in acidic conditions or high temperatures; therefore, it is difficult to use enantiopure R-LA as a pharmaceutical or nutraceutical ingredient. Recently, we have prepared stabilized R-LA through complex formation with several types of cyclodextrins and shown that the complex with γ-cyclodextrin yielding R-LA/CD is most promising [23,24,25,26].

Cyclodextrins (CDs), cyclic oligosaccharides consisting of d-glucopyranose, are widely used to improve the solubility and stability of lipophilic molecules including R-LA [23,24,25]. It has been recently shown that complexation of R-LA with γ-CD significantly improved the bioavailability of R-LA in rats by enhancing intestinal absorption [26]. However, there are no reports of the pharmacokinetic profile of the stabilized R-LA/CD in human subjects. Takahashi *et al.* [27] prepared three types of racemic LA/CD complexes including γ-CD and measured the plasma LA concentration after a single oral administration of 600 mg LA in healthy humans. However, their study design was not suitable for pharmacokinetic studies of LA because the first blood sampling point was 30 min after dosing, which could be too late to evaluate the maximum plasma concentration (*C*_max_).

It is well documented in the literature that 600 mg of racemic LA has been dosed in pharmacokinetic or pharmacology studies [28,29,30]. In many clinical trials, racemic LA has been administered at doses between 600 and 1800 mg/day to diabetic patients [6,8,31,32]. Breithaupt-Grögler *et al.* [29] monitored the plasma LA concentration 10 min after dosing and demonstrated that the area under the plasma concentration–time curve (AUC) values showed dose linearity after single doses of 50 to 600 mg LA in the fasted volunteers. They also showed that there were no drug-induced effects in healthy male subjects. Gleiter *et al.* [28] showed that the food intake influenced the bioavailability of LA and that the mean plasma LA concentration of the fed healthy volunteers who received a single 600-mg dose of racemic LA was lower than that of the fasted healthy volunteers. Gleiter *et al.* and Breithaupt-Grögler *et al.* [28,29] also reported in 1996 and 1999, respectively, that the bioavailability of the R-LA enantiomer was higher than that of S-LA when a racemic mixture of LA (R-LA:S-LA = 50:50) was administrated. Our recent animal study showed similar enantioselective pharmacokinetics of LA [33]. Carlson *et al.* [34] conducted a clinical study to determine the pharmacokinetics of R-LA in healthy human subjects administered 600 mg of R-LA as its sodium salt in 2007, when enantiopure R-LA became available. This was the first reported study to demonstrate the pharmacokinetics of enantiopure R-LA. Since then, several pharmacokinetic clinical studies of R-LA in healthy subjects have been conducted, primarily in Germany [35] and the United States [34], but there has been no scientific research conducted in Japanese subjects.

The aim of this study was to evaluate the bioavailability of R-LA/CD in healthy human subjects and analyzed the pharmacokinetics of the plasma R-LA levels after a single oral administration of 600 mg of R-LA or 6 g of R-LA/CD (equivalent amount of 600 mg of R-LA).

## 2. Results

### 2.1. Subjects’ Characteristics, Safety, and Tolerability

The subjects’ characteristics are shown in Table 1. All six subjects successfully completed both phases of the study, and the plasma samples were used for drug analysis with the plasma glucose level assay. LA has been shown to stimulate glucose uptake, via both glucose transporter type 4 (GLUT4) translocation and GLUT4 activation, in 3T3-L1 adipocytes and L6 myotubes [36] and via activation two important molecules of the insulin signaling pathway-insulin receptor substrate-1 (IRS-1) protein and phosphatidylinositor 3-kinase (PI 3-kinase) [36]. Previously, Kamenova [9] measured the fasting plasma glucose levels of type 2 diabetic patients before and after 4 weeks of treatment with 600 mg LA twice daily and compared them with that of non-diabetic subjects in Bulgaria, and no significant changes of plasma glucose level were observed. However, there was a possibility that a single oral administration of 6 g of R-LA/CD induced an acute change of blood glucose level in healthy Japanese subjects. In addition, there was a risk that R-LA would lower the blood glucose level markedly in the healthy subjects if the bioavailability of R-LA was improved through the complexation with CD. Therefore, the plasma glucose level was measured for the evaluation of safety and tolerability. As a result, the plasma glucose level did not change throughout the study (Table 2). There were no serious adverse drug reactions or side effects reported by the participants or observed by investigators during the study periods.

### 2.2. Plasma Pharmacokinetics of R-α-Lipoic Acid (R-LA)

**Figure 2:**
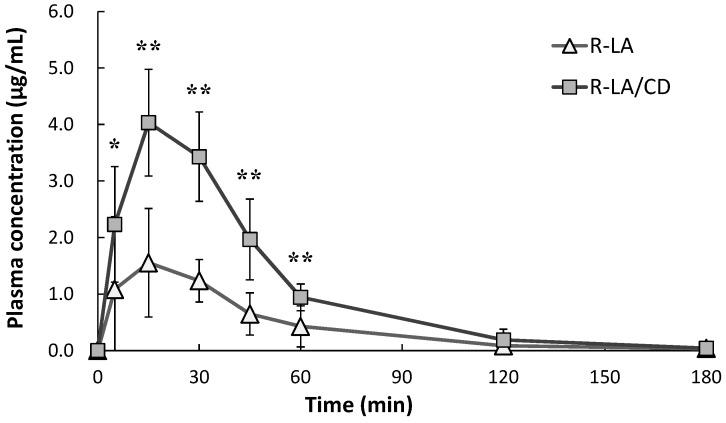
Plasma concentrations of R-α-lipoic acid following a single oral 600-mg dose of R-α-lipoic acid or 6 g of R-α-lipoic acid/γ-cyclodextrin in healthy volunteers. Values are the mean ± S.D. from six subjects. * *p* < 0.05 and ** *p* < 0.01; R-LA/CD (R-α-lipoic acid/γ-cyclodextrin); R-LA (R-α-lipoic acid); Note: 6 g of R-LA/CD is the equivalent amount of 600 mg of R-LA.

The mean plasma R-LA concentration *versus* time profiles after oral administration of R-LA or R-LA/CD is shown in Figure 2. The pharmacokinetic (PK) parameters are listed in Table 3 and Table 4. For all subjects, the plasma concentrations of R-LA after a single oral dose of R-LA/CD were significantly higher than those of R-LA at 5, 15, 30, 45, and 60 min after oral administration. At each time point measured, the mean plasma concentrations of R-LA after an oral dose of R-LA/CD were higher than those of R-LA. The mean *C*_max_ values were 1.68 ± 1.01 and 4.10 ± 0.96 µg/mL (mean ± S.D.) for R-LA and R-LA/CD administration, respectively. The *C*_max_ value of R-LA/CD was significantly higher than that of R-LA. The mean area under the plasma concentration–time curve from time zero to the last sampling time (AUC_0–180min_) values were 78.0 ± 43.5 and 195.9 ± 17.7 µg·min/mL (mean ± S.D.) for R-LA and R-LA/CD administration, respectively. The mean AUC_0–180min_ of R-LA/CD was 2.5 times higher than that of R-LA and was also statistically significant. The time to reach maximum plasma concentration (*T*_max_) and the half-life (*T*_1/2_) were not different between R-LA and R-LA/CD. At 180 min, the mean plasma R-LA concentrations had nearly returned to base line levels for both R-LA and R-LA/CD.

## 3. Discussion

In this study, we investigated the pharmacokinetics of R-LA in healthy Japanese subjects administered enantiopure non-complexed R-LA and stabilized R-LA/CD. This is the first study to show the plasma R-LA profile after a single administration of R-LA/CD in healthy human subjects. For all subjects, the plasma concentrations of R-LA after oral administration of R-LA/CD were significantly higher than those after a single dose of R-LA at 5, 15, 30, 45, and 60 min. Therefore, the calculated *C*_max_ value and AUC_0–180min_ of R-LA/CD were significantly higher than those of R-LA. The highest *C*_max_ we found was 5.08 µg/mL in Subject 3, which is lower than the highest *C*_max_ of 33.8 µg/mL (mean *C*_max_ was 16.0 µg/mL) reported by Carlson *et al.* [34]. In their clinical trial, R-LA sodium salt was administered; in our study, we administered the free acid form of R-LA. The mean *T*_1/2_ of R-LA sodium salt in Carlson’s study was 14.0 min, and those of R-LA and R-LA/CD in our study were 17.5 and 23.3 min, respectively. The differences in *C*_max_ and *T*_1/2_ among the two studies may be explained by the greater solubility of R-LA sodium salt *versus* the free acid form. As Carlson *et al.* [34] reported, the PK values for R-LA were widely varying. The results of this study indicate that the bioavailability of R-LA/CD is certainly higher than that of non-complexed R-LA. Differences in the bioavailability of the R-LA and R-LA/CD are possibly attributed to differences in their stabilities. We previously evaluated the physico-chemical properties and the stability of R-LA/CD and showed that R-LA/CD is much more stable against low pH or heat than non-complexed R-LA *in vitro* [23]. Our group also reported that R-LA was effectively absorbed from the small intestine when orally administered to rats as R-LA/CD [26]. This indicated that R-LA/CD was stable in the stomach and could be easily dissolved in the lumen of the rat intestine. The mean AUC of R-LA for rats after a single oral administration of R-LA/CD was 2.2 times higher than that of non-complexed R-LA [26]. In this study, the mean AUC_0–180min_ of R-LA/CD was 2.5 times higher than that of R-LA in healthy human subjects. pH value in the stomach of fasted human is around 1.2 and generally lower than that of rat. Our previous *in vitro* study showed that R-LA/CD was stable at low pH (~2), as found in the human stomach [23]. In the same report, we also showed that non-complexed R-LA was unstable (43% of the R-LA treated with low pH solution remained). Our results indicated that R-LA/CD is stable in the human stomach because of the stability of R-LA/CD. In our previous studies on the PK of R-LA/CD in rats [26], 20 mg of R-LA/kg was administrated with a feeding needle. The resulted mean C_max_ values were 1.7 ± 0.9 and 3.4 ± 2.5 µg/mL, and the mean AUC_0–120min_ values were 56 ± 35 and 121 ± 24 µg·min/mL (mean ± S.D.) for R-LA and R-LA/CD administration, respectively. Although the doses were different among the two studies, the PK values were similar. The species were different for the two studies on the PK of R-LA/CD in humans and rats, and the absorbable amounts of R-LA should be different. Therefore, for the new developed neutraceuticals, the PK of them should be analyzed not only in animals but also in humans.

The present study showed that CD complexation improved the bioavailability of R-LA in healthy human volunteers, which could be caused by the stability of R-LA/CD. We observed no changes in *T*_max_ and *T*_1/2_ in response to a single oral dose of R-LA or R-LA/CD. Improvement of the oral bioavailability through complexation with CDs were reported in previous studies on a variety of drugs in rabbits (e.g., tolbutamide/CD complexes) [37] and humans (coenzyme Q10/CD complex) [38] and it was reviewed thoroughly by Carrier *et al.* [39] and Loftsson *et al.* [40]. Tolbutamide and coenzyme Q10 (CoQ10) are practically insoluble in water; the bioavailability of them is therefore very low. Complexation with CDs enhanced the solubility or dispersibility of these insoluble drugs and improved their bioavailability. For the case of tolbutamide, *T*_max_ and *T*_1/2_ changed through the complexation with CD. For the case of CoQ10, there was no statistically difference in *T*_max_ and *T*_1/2_, but the profile of plasma CoQ10 level changed. For the case of R-LA/CD, R-LA is soluble in both aqueous and non-aqueous media. In this study, the absorption of R-LA was rapid and CD complexation did not affect the plasma R-LA level profile *T*_max_ and *T*_1/2_. The original physico-chemical property of the guest drugs could affect the pharmacokinetics of the drugs/CD complexes. These results indicate that CD complexation should be a promising technology for both insoluble and unstable but bioactive compounds.

It should be noted that only male subjects were enrolled in the present study. However, as reported previously by Carlson *et al.* [34], there were no statistical differences between the pharmacokinetic data for R-LA from males and females. Thus, we consider that the effect of CD complexation on the bioavailability of R-LA in females would be comparable to that in males as the present study. The number of human subjects participated in this study was 6, which was not large number, even though the results were statistically significant. We calculated sample size with the software “EZR” [41] on the assumption that the same trial would be planned in a larger size based on these results (statistical power = 0.8, error = 0.05, two sided), and the calculated size was *n* = 4, indicating that the number of subjects in this study was sufficient.

## 4. Materials and Methods

### 4.1. Subjects and Study Design

The study was performed according to the Declaration of Helsinki and with the permission of the independent ethics committee (the Ethics Committee at Kobe University Graduate School, code; 1424, approval date; 17 May 2013). All participants were recruited according to the inclusion criteria, which comprised being between an age of 20 to 45, being in good health and physical condition, and having a demonstrated ability to read, understand, and sign the informed consent form. The exclusion criteria included significant clinical deviation from normal as determined by investigators, a history or suspicion of diabetes, hypoglycemia, or thiamine deficiency disease.

A total of six men between 31 and 34 years old (age: 33.0 ± 1.3 years old, body weight: 61.8 ± 6.4 kg; mean ± S.D.) in good health and physical condition as determined by a review of their medical histories and an interview were admitted to the study after providing their informed written consent.

The study was an open-label, randomized-sequence, single-dose, two-way crossover study with a wash-out period of at least 2 weeks in healthy volunteers. In the wash-out period, the subjects did not take any nutritional supplements such as vitamins. After 12 h of fasting, a single oral 600-mg dose of R-LA or 6 g of R-LA/CD (the equivalent amount of R-LA), each of which was in powder form, was administrated with 200 mL of plain water. R-LA and R-LA/CD were supplied by CycloChem Bio, Co., Ltd. (Kobe, Japan). Enantiomeric excess of R-LA was 99.4%, which were measured by high performance liquid chromatography (HPLC) as described before [23]. R-LA/CD was prepared as follows. R-LA sodium salt was dissolved in water, and the corresponding molar amount of γ-CD for a 1:1 ratio with R-LA sodium salt was added. The solution was mixed and adjusted pH by adding 1 M HCl. Then, the suspension was continuously stirred in the dark and freeze-dried. R-LA content in R-LA/CD was 10%. R-LA exists as an acid form in R-LA/CD. Food and drinks were withheld from the participants during the study, except for plain water. The blood samples were collected by withdrawing 5 mL from the antecubital vein at 0 (pre-dose), 5, 15, 30, 45, 60, 120, and 180 min after oral administration. In total, 45 mL of blood was taken from each subject. The collected blood samples were immediately centrifuged at 1500× *g* for 10 min, and the plasma fractions were transferred into new tubes. They were stored at −80 °C until further analysis.

### 4.2. Drug Analysis and Pharmacokinetics

Plasma concentrations of R-LA were determined using an API 3200™ (AB SCIEX, Framingham, MA, USA) liquid chromatography coupled with tandem mass spectrometry (LC-MS/MS) system interfaced with a Shimadzu Prominence HPLC system (Shimadzu, Kyoto, Japan) as previously described [26]. Since, in plasma, LA is bound to proteins, our protocol used acetonitrile to release LA so that total LA (sum of protein-bound and free LA) was measured. The reported baseline plasma level of LA, which was released by enzymatic treatment, was in the range 1–25 ng/mL [42] or 1 ng/mL or less [43]. Carlson *et al.* [34] also reported that the baseline R-LA was 0.05–0.25 µg/mL in the subject who were regular users of R-LA. In the present study, total R-LA in plasma was measured by LC-MS/MS assuming that the endogenous LA was negligible. All reagents used were of analytical grade.

*C*_max_, *T*_max_, AUC_0–180min_, and *T*_1/2_ were determined for each individual. *C*_max_ and *T*_max_ were obtained directly from individual plasma concentration–time profiles, while AUC_0–180min_ was calculated using the trapezoidal method. *T*_1/2_ was calculated using the logarithmic transformed plasma concentration values of the individual using the macro program MOMENT (EXCEL) [44]. To minimize Akaike’s information criterion, which was incorporated in the macro program, the last three or four points of the plasma concentration were used for linear regression.

### 4.3. Plasma Glucose Analysis

The plasma glucose level of the plasma sample after oral administration of R-LA/CD or R-LA was measured using the Glucose CII-Test Wako kit (Wako Pure Chemical Industries, Ltd., Osaka, Japan) according to the protocol.

### 4.4. Tolerability

Vital signs were observed at pre-dose and throughout the study. Subjects were asked to report any discomfort or adverse events at any time during the study period.

### 4.5. Statistical Analysis

All data are expressed as the mean ± S.D. Statistical analysis was based on the Student’s *t*-test for *T*_max_ and *T*_1/2_. For AUC_0–180min_ and *C*_max_, the Student’s *t*-test was conducted after logarithmic transformation of the data. The statistical analysis was performed using Pharmaco Analyst II (Scientist Press Co., Ltd., Tokyo, Japan) and Ekuseru-Toukei (Social Survey Research Information Co., Ltd., Tokyo, Japan). Values of *p* < 0.05 or *p* < 0.01 were considered statistically significant and are indicated with asterisks (*) or (**), respectively.

## 5. Conclusions

R-LA/CD can be easily absorbed in the intestine. The mean AUC_0–180min_ of R-LA in the subjects orally administered 6 g of R-LA/CD (equivalent amount of 600 mg of R-LA) was 2.5 times higher than that of the subjects administered 600 mg of R-LA. Thus, our results suggest that complexation of R-LA with γ-CD significantly enhance its bioavailability in healthy human volunteers, making it a promising technology for delivering functional but unstable ingredients such as R-LA. Additionally, there were no drug-induced side effects observed. These results indicate that 6 g of R-LA/CD is suitable for nutraceutical purposes.

## Figures and Tables

**Table 1:** Characteristics of the six subjects.

Subject	Gender	Age	Body Weight (kg)	Body Mass Index (kg/m^2^)
1	Male	31	68	22.2
2	Male	34	55	21.5
3	Male	33	54	19.1
4	Male	32	67	23.7
5	Male	34	67	26.5
6	Male	34	60	20.8
Mean ± S.D.		33.0 ± 1.3	61.8 ± 6.4	22.3 ± 2.6

**Table 2:** Plasma glucose level in the subjects after a single oral administration of 600 mg of R-LA or 6 g of R-LA/CD.

Time (min)	Plasma Glucose (mg/dL)	
	R-LA	R-LA/CD
0	78 ± 7	82 ± 12
5	80 ± 10	79 ± 11
15	77 ± 7	80 ± 11
30	80 ± 9	78 ± 10
45	79 ± 8	77 ± 8
60	78 ± 10	76 ± 10
120	79 ± 11	82 ± 12
180	77 ± 12	80 ± 12

Values are the mean ± S.D. from six subjects. R-LA/CD (R-α-lipoic acid/γ-cyclodextrin); R-LA (R-α-lipoic acid); Note: 6 g of R-LA/CD is the equivalent amount of 600 mg of R-LA.

**Table 3:** Pharmacokinetic parameters of individual subjects administrated with a single oral 600-mg dose of R-α-lipoic acid or 6 g of R-α-lipoic acid/γ-cyclodextrin.

Subject	R-LA		R-LA/CD	
	*C*_max_ (µg/mL)	AUC_0–180min_ (µg·min/mL)	*C*_max_ (µg/mL)	AUC_0–180min_ (µg·min/mL)
1	1.02	43.6	2.62	186.7
2	1.03	46.7	4.45	214.7
3	1.83	74.9	5.08	216.7
4	1.02	53.8	4.93	197.3
5	3.62	158.8	4.23	189.4
6	1.55	90.5	3.30	170.5

R-LA/CD (R-α-lipoic acid/γ-cyclodextrin); R-LA (R-α-lipoic acid); *C*_max_ (maximum plasma concentration); AUC (area under the plasma concentration–time curve).

**Table 4:** Pharmacokinetic parameters for subjects orally administered with 600 mg of R-α-lipoic acid or 6 g of R-α-lipoic acid/γ-cyclodextrin.

Property	R-LA	R-LA/CD
*C*_max_ (µg/mL)	1.68 ± 1.01	4.10 ± 0.96 **
AUC_0–18 min_ (µg·min/mL)	78.0 ± 43.5	195.9 ± 17.7 **
*T*_max_ (min)	20.8 ± 10.7	17.5 ± 6.1
*T*_1/2_ (min)	38.9 ± 12.2	23.3 ± 10.3

Values are the mean ± S.D. from six subjects, ** *p* < 0.01; R-LA/CD (R-α-lipoic acid/γ-cyclodextrin); R-LA (R-α-lipoic acid); *C*_max_ (maximum plasma concentration); AUC (area under the plasma concentration–time curve); *T*_max_ (Time to the maximum plasma concentration); *T*_1/2_ (half-life)

## References

[1] Packer L., Kraemer K., Rimbach G. (2001). Molecular aspects of lipoic acid in the prevention of diabetes complications. Nutrition.

[2] Khanna S., Roy S., Packer L., Sen C.K. (1999). Cytokine-induced glucose uptake in skeletal muscle: Redox regulation and the role of α-lipoic acid. Am. J. Physiol..

[3] Bramanti V., Tomassoni D., Bronzi D., Grasso S., Currò M., Avitabile M., Li Volsi G., Renis M., Ientile R., Amenta F. (2010). α-Lipoic acid modulates GFAP, vimentin, nestin, cyclin D1 and MAP-kinase espression in astroglial cell cultures. Neurochem. Res..

[4] Kramer K., Packer L., Hoppe P. (2001). R-α-lipoic acid. Nutraceuticals in Health and Disease Prevention.

[5] Grasso S., Bramanti V., Tomassoni D., Bronzi D., Malfa G., Traini E., Napoli M., Renis M., Amenta F., Avola R. (2014). Effect of lipoic acid and α-glyceryl-phosphoryl-choline on astroglial cell proliferation and differentiation in primary culture. J. Neurosci. Res..

[6] Ziegler D., Hanefeld M., Ruhnau K.J., Hasche H., Lobisch M., Schütte K., Kerum G., Malessa R. (1999). Treatment of symptomatic diabetic polyneuropathy with the antioxidant α-lipoic acid: A 7-month multicenter randomized controlled trial (ALADIN III Study). ALADIN III study group. α-lipoic acid in diabetic neuropathy. Diabetes Care.

[7] Haak E.S., Usadel K.H., Kohleisen M., Yilmaz A., Kusterer K., Haak T. (1999). The effect of α-lipoic acid on the neurovascular reflex arc in patients with diabetic neuropathy assessed by capillary microscopy. Microvasc. Res..

[8] Ziegler D., Ametov A., Barinov A., Dyck P.J., Gurieva I., Low P.A., Munzel U., Yakhno N., Raz I., Novosadova M. (2006). Oral treatment with α-lipoic acid improves symptomatic diabetic polyneuropathy the SYDNEY 2 trial. Diabetes Care.

[9] Kamenova P. (2006). Improvement of insulin sensitivity in patients with type 2 diabetes mellitus after oral administration of α-lipoic acid. Hormones.

[10] Chang J.W., Lee E.K., Kim T.H., Min W.K., Chun S., Lee K.-U., Kim S.B., Park J.S. (2007). Effects of α-lipoic acid on the plasma levels of asymmetric dimethylarginine in diabetic end-stage renal disease patients on hemodialysis: A pilot study. Am. J. Nephrol..

[11] Gębka A., Serkies-Minuth E., Raczyńska D. (2014). Effect of the administration of α-lipoic acid on contrast sensitivity in patients with type 1 and type 2 diabetes. Med. Inflamm..

[12] Morcos M., Borcea V., Isermann B., Gehrke S., Ehret T., Henkels M., Schiekofer S., Hofmann M., Amiral J., Tritschler H. (2001). Effect of α-lipoic acid on the progression of endothelial cell damage and albuminuria in patients with diabetes mellitus: An exploratory study. Diabetes Res. Clin. Pract..

[13] El-Nabarawy S.K., Mohamed M.A., Ahmed M.M., El-Arabi G.H. (2011). α-Lipoic acid ameliorates the oxidative status and serum iron in diabetic patients. J. Pharm. Biomed. Sci..

[14] Huang E.A., Gitelman S.E. (2008). The effect of oral α-lipoic acid on oxidative stress in adolescents with type 1 diabetes mellitus. Pediatr. Diabetes.

[15] Hager K., Marahrens A., Kenklies M., Riederer P., Münch G. (2001). α-Lipoic acid as a new treatment option for Alzheimer type dementia. Arch. Gerontol. Geriatr..

[16] Maczurek A., Hager K., Kenklies M., Sharman M., Martins R., Engel J., Carlson D.A., Münch G. (2008). Lipoic acid as an anti-inflammatory and neuroprotective treatment for Alzheimer’s disease. Adv. Drug Deliv. Rev..

[17] Hager K., Kenklies M., McAfoose J., Engel J., Münch G. (2007). α-Lipoic acid as a new treatment option for Alzheimer’s disease—A 48 months follow-up analysis. J. Neural Transm..

[18] Bustamante J., Lodge J.K., Marcocci L., Tritschler H.J., Packer L., Rihn B.H. (1998). α-Lipoic acid in liver metabolism and disease. Free Radic. Biol. Med..

[19] Vincent H.K., Bourguignon C.M., Vincent K.R., Taylor A.G. (2007). Effects of α-lipoic acid supplementation in peripheral arterial disease: A pilot study. J. Altern. Complement. Med..

[20] Huerta A.E., Navas-Carretero S., Prieto-Hontoria P.L., Martínez J.A., Moreno-Aliaga M.J. (2015). Effects of α-lipoic acid and eicosapentaenoic acid in overweight and obese women during weight loss. Obesity.

[21] Koh E.H., Lee W.J., Lee S.A., Kim E.H., Cho E.H., Jeong E., Kim D.W., Kim M.-S., Park J.-Y., Park K.-G. (2011). Effects of α-lipoic acid on body weight in obese subjects. Am. J. Med..

[22] Carbonelli M.G., Renzo L.D., Bigioni M., Daniele N.D., Lorenzo A.D., Fusco M.A. (2010). α-Lipoic acid supplementation: A tool for obesity therapy?. Curr. Pharm. Des..

[23] Ikuta N., Sugiyama H., Shimosegawa H., Nakane R., Ishida Y., Uekaji Y., Nakata D., Pallauf K., Rimbach G., Terao K. (2013). Analysis of the enhanced stability of R(+)-α lipoic acid by the complex formation with cyclodextrins. Int. J. Mol. Sci..

[24] Ikuta N., Tanaka A., Otsubo A., Ogawa N., Yamamoto H., Mizukami T., Arai S., Okuno M., Terao K., Matsugo S. (2014). Spectroscopic studies of R(+)-α-lipoic acid—Cyclodextrin complexes. Int. J. Mol. Sci..

[25] Ikuta N., Endo T., Hosomi S., Setou K., Tanaka S., Ogawa N., Yamamoto H., Mizukami T., Arai S., Okuno M. (2015). Structural analysis of crystalline R(+)-α-lipoic acid-α-cyclodextrin complex based on microscopic and spectroscopic studies. Int. J. Mol. Sci..

[26] Uchida R., Iwamoto K., Nagayama S., Miyajima A., Okamoto H., Ikuta N., Fukumi H., Terao K., Hirota T. (2015). Effect of γ-cyclodextrin inclusion complex on the absorption of R-α-lipoic acid in rats. Int. J. Mol. Sci..

[27] Takahashi H., Kishino E., Mikuni K., Kiuchi Y., Beppu H., Okazaki H., Shimpo K., Sonoda S. (2012). Effect of different types of cyclodextrins on gastrointestinal absorption of α-lipoic acid in rats and humans. J. Appl. Glycosci..

[28] Gleiter C.H., Schung B.S., Hermann R., Elze M., Blume H.H., Gundert-Remy U. (1996). Influence of food intake on the bioavailability of thioctic acid enantiomers. Eur. J. Pharmacol..

[29] Breithaupt-Grögler K., Niebch G., Schneider E., Erb K., Hermann R., Blume H.H., Schug B.S., Belz G.G. (1999). Dose-proportionality of oral thioctic acid—Coincidence of assessments via pooled plasma and individual data. Eur. J. Pharm. Sci..

[30] Teichert J., Tuemmers T., Achenbach H., Preiss C., Hermann R., Ruus P., Preiss R. (2005). Pharmacokinetics of α-lipoic acid in subjects with severe kidney damage and end-stage renal disease. J. Clin. Pharmacol..

[31] Ziegler D., Schatz H., Conrad F., Gries F.A., Ulrich H., Reichel G. (1997). Effects of treatment with the antioxidant α-lipoic acid on cardiac autonomic neuropathy in NIDDM patients. A 4-month randomized controlled multicenter trial (DEKAN study). Diabetes Care.

[32] Reljanovic M., Reichel G., Rett K., Lobisch M., Schuette K., Moller W., Tritschler H.J., Mehnert H. (1999). Treatment of diabetic polyneuropathy with the antioxidant thioctic acid (α-lipoic acid): A two year multicenter randomized double-blind placebo-controlled trial (ALADIN II). Free Radic. Res..

[33] Uchida R., Okamoto H., Ikuta N., Terao K., Hirota T. (2015). Enantioselective pharmacokinetics of α-lipoic acid in rats. Int. J. Mol. Sci..

[34] Carlson D.A., Smith A.R., Fischer S.J., Young K.L., Packer L. (2007). The plasma pharmacokinetics of R-(+)-lipoic acid administrated as sodium R-(+)-lipoate to healthy human subjects. Altern. Med. Rev..

[35] Hermann R., Niebch G., Borbe H.O., Fieger-Büschges H., Ruus P., Nowak H., Riethmüller-Winzen H., Peukert M., Blume H. (1996). Enantioselective pharmacokinetics and bioavailability of different racemic α-lipoic acid formulations in healthy volunteers. Eur. J. Pharm. Sci..

[36] Konrad D., Somwar R., Sweeney G., Yaworsky K., Hayashi M., Ramlal T., Klip A. (2001). The antihyperglycemic drug α-lipoic acid stimulates glucose uptake via both GLUT4 translocation and GLUT4 activation: Potential role of p38 mitogen-activated protein kinase in GLUT4 activation. Diabetes.

[37] Veiga F., Fernandes C., Teixeira F. (2000). Oral bioavailability and hypoglycaemic activity of tolbutamide/cyclodextrin inclusion complexes. Int. J. Pharm..

[38] Terao K., Nakata D., Fukumi H., Schmid G., Arima H., Hirayama F., Uekama K. (2006). Enhancement of oral bioavailability of coenzyme Q10 by complexation with γ-cyclodextrin in healthy adults. Nutr. Res..

[39] Carrier R.L., Miller L.A., Ahmed I. (2007). The utility of cyclodextrins for enhancing oral bioavailability. J. Control Release.

[40] Loftsson T., Moya-Ortega M.D., Alvarez-Lorenzo C., Concheiro A. (2015). Pharmacokinetics of cyclodextrins and drugs after oral and parenteral administration of drug/cyclodextrin complexes. J. Pharm. Pharmacol..

[41] Kanda Y. (2013). Investigation of the freely available easy-to-use software “EZR” for medical statistics. Bone Marrow Transplant..

[42] Teichert J., Preiss R. (1992). HPLC-methods for determination of lipoic acid and its reduced form in human plasma. Int. J. Clin. Pharmacol. Ther. Toxicol..

[43] Teichert J., Preiss R. (2002). High-performance liquid chromatographic assay for α-lipoic acid and five of its metabolites in human plasma and urine. J. Chromatogr. B Anal. Technol. Biomed. Life Sci..

[44] Tabata K., Yamaoka K., Kaibara A., Suzuki S., Terakawa M., Hata T. (1999). Moment analysis program available on Microsoft Excel^®^. Drug Metab. Pharmacokinet..

